# A Computer Vision Approach to Identifying Ticks Related to Lyme Disease

**DOI:** 10.1109/JTEHM.2021.3137956

**Published:** 2021-12-30

**Authors:** Sina Akbarian, Mark P. Nelder, Curtis B. Russell, Tania Cawston, Laurent Moreno, Samir N. Patel, Vanessa G. Allen, Elham Dolatabadi

**Affiliations:** Public Health Ontario 153300 Toronto ON M5G 1M1 Canada; Vector Institute for Artificial Intelligence Toronto ON M5G 1M1 Canada; Enteric, Zoonotic and Vector-Borne Diseases, Health Protection, Operations and ResponsePublic Health Ontario 153300 Toronto ON M5G 1M1 Canada; Public Health LaboratoriesPublic Health Ontario 153300 Sault Ste. Marie ON P6B 0A9 Canada; Innovations and Partnerships OfficeUniversity of Toronto 7938 Toronto ON M5S 1A1 Canada; Department of Laboratory Medicine and PathobiologyUniversity of Toronto 7938 Toronto ON M5S 1A1 Canada; Medical MicrobiologyPublic Health Ontario 153300 Toronto ON M5G 1M1 Canada; Institute of Health Policy, Management and Evaluation, University of Toronto 7938 Toronto ON M5S 1A1 Canada

**Keywords:** Computer vision, convolution neural network, infectious disease, *Ixodes scapularis*, knowledge transfer, Lyme disease, public health, surveillance, vector-borne disease

## Abstract

*Background:* Lyme disease (caused by *Borrelia burgdorferi*) is an infectious disease transmitted to humans by a bite from infected blacklegged ticks (*Ixodes scapularis*) in eastern North America. Lyme disease can be prevented if antibiotic prophylaxis is given to a patient within 72 hours of a blacklegged tick bite. Therefore, recognizing a blacklegged tick could facilitate the management of Lyme disease. *Methods:* In this work, we build an automated detection tool that can differentiate blacklegged ticks from other tick species using advanced computer vision approaches in real-time. Specially, we use convolution neural network models, trained end-to-end, to classify tick species. Also, advanced knowledge transfer techniques are adopted to improve the performance of convolution neural network models. *Results:* Our best convolution neural network model achieves 92% accuracy on unseen tick species. *Conclusion:* Our proposed vision-based approach simplifies tick identification and contributes to the emerging work on public health surveillance of ticks and tick-borne diseases. In addition, it can be integrated with the geography of exposure and potentially be leveraged to inform the risk of Lyme disease infection. This is the first report of using deep learning technologies to classify ticks, providing the basis for automation of tick surveillance, and advancing tick-borne disease ecology and risk management.

## I. Introduction

Lyme disease is caused by the spirochete *Borrelia burgdorferi* sensu stricto and is spread to humans through the bite of an infected blacklegged tick (*Ixodes scapularis*) in most of eastern North America. Lyme disease is the most common tick-borne disease in North America, and in the United States (US), approximately 475,000 cases occur per year (2010–2018), with about 35,000 of these reported through reportable disease surveillance [1], [2]. In Canada, Lyme disease is an emerging infectious disease, increasing from 992 cases in 2016 to 2636 cases in 2019 [3]. The increase in Lyme disease incidence is associated with the expanding range of the blacklegged tick, driven in part by climate change; i.e., an increase in annual cumulative degree days above 0°C [4].

Lyme disease generally begins with influenza-like symptoms such as arthralgia, chills, fever, myalgia, and stiff neck, with the appearance of an erythema migrans rash 2 to 30 days after a bite from an infectious blacklegged tick [5]–[7]. Treatment with antibiotics typically clears the *B. burgdorferi* infection; however, if left untreated, infection can progress to disseminated disease with higher chances of morbidity, long-term sequelae and post-treatment Lyme disease syndrome [6], [8]–[11]. Lyme disease can be prevented if antibiotic prophylaxis is given to a patient within 72 hours of a tick bite [11].

Understanding where blacklegged ticks and *B. burgdorferi* co-occur is vital to informing healthcare providers and the public about the risks of Lyme disease. Throughout North America, blacklegged tick surveillance is patchy (using various passive and active tools) or sometimes absent. Passive surveillance involves submissions of ticks from the healthcare providers or the public for identification and/or detection of *B. burgdorferi* [12]. Another passive technique is through the submission of tick pictures to web-based applications for identification, where an expert manually identifies each tick picture submission (e.g., eTick.ca in Canada). Active surveillance includes tick dragging from the environment or live-animal trapping. Active and passive surveillance are designed to monitor tick populations and are not designed with a clinical application in mind; therefore, additional tools are needed to aide clinician management of Lyme disease. In addition, these surveillance techniques are time-consuming, logistically challenging and costly to operate and maintain. In a clinical setting, healthcare providers require a faster answer to whether a tick from a patient is a blacklegged tick or another tick pest species. Healthcare providers and patients would benefit with a quicker assessment of what species of tick was involved as this could help with decisions of whether to monitor a patient’s symptoms or to provide antibiotic prophylaxis. This quicker turnaround time would be especially beneficial in areas where blacklegged ticks are expanding and not easily recognized.

Rapid automatic identification of blacklegged ticks using machine learning technologies is a potential solution in mitigating some of the challenges of the current tick identification process, leading to automation of tick surveillance. Recent advances in machine learning and deep learning have contributed to the prevention, management, and surveillance of infectious diseases [13]–[16]. Here we built computer vision models - enabled by advanced deep neural networks - to automatically identify blacklegged ticks from other tick species. To facilitate adoption and future potential implementation of this technology into a real-life environment, we developed a web application that can be further used in prospective validation of the model by healthcare stakeholders and the public. Here we present our initial proof-of-concept work on using computer vision models to identify blacklegged ticks.

## II. Materials and Methods

### A. Background

Automation of surveillance and identification of tick species using computer vision models could be potentially powered by advances in deep neural networks [17]. Convolution neural network (CNN) is a class of deep neural networks that is most commonly applied to computer vision tasks. However, training CNN models from scratch is not without complications and requires collection and annotation of large data sets which limit their utilization in health settings [18]. A popular approach for handling this shortcoming is to leverage the “transferability” of knowledge embedded in the pre-trained CNNs and to transfer that knowledge from a known source task to a new target task [19]–[21]. The most widely used approach in knowledge transfer is transfer learning where a source deep neural network is first trained with a large dataset such as ImageNet [22] (this is called pre-training) and then the networks’ learned weights (knowledge) are used as an initialization to train a target deep neural network on a smaller dataset such as medical images (this is called fine-tuning) [23], [24]. Fine-tuning requires minimal modifications where some of the network parameters remain frozen during training [24], [25]. Transfer learning has been applied successfully to various computer vision tasks such as object classification and feature generation for both generic and medical domains [26]–[28]. However, depending on the problem, transfer learning might not be the best approach and provide no benefit especially if the source and target domains are semantically and substantially different [17]. An alternative to transfer learning for heterogeneous domains is another knowledge transfer technique which is called the teacher-student learning framework. Knowledge transfer in teacher-student learning framework occurs between two distinct networks namely a teacher network and student network where the student network is trained to imitate the output of a more extensive and powerful teacher network or ensemble of teacher networks [19], [20]. One of the popular teacher-student training frameworks is attention transfer proposed by Zagoruyko *et al.* [29]. In this method, the teacher’s feature maps guide the student to learn data patterns. Using this approach, given the attention maps of a teacher network, the student network is trained to imitate the exact behavior of the teacher network by trying to replicate its output at a layer receiving attention from the teacher.

In this work, we built our computer vision pipeline using different knowledge transfer approaches (e.g., attention transfer) [29], [30] due to the small size (several thousand images) of our tick data set. Also, our tick data set included several noisy and blurry images due to the presence of very small nymphal ticks, so we utilized Label Smoothing Regularization (LSR) [21] besides attention transfer as a regularizer to enhance CNN models’ robustness and generalization. LSR converts one-hot encoded labels (hard labels) to soft labels with a mixture of uniform distribution. In addition to model improvement, both attention transfer and LSR provide benefit to model compression [20], [31] which enables the deployment of CNN models on mobile phones or website applications.

### B. Data Set Description

**FIGURE 1.**
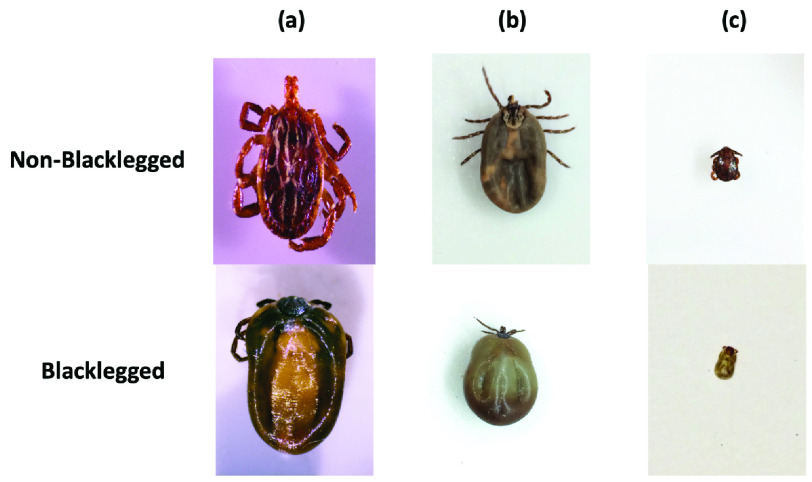
(a) High-resolution microscopic images, (b) Mobile phone images of fully engorged ticks, and (c) Mobile phone images of unfed ticks. Fully and slightly engorged ticks can triple in volume when filled with blood.

Our tick data set was collected from May 2019 to November 2019 by Public Health Ontario, which includes images of blacklegged and over 6 other non-blacklegged species such as the American dog tick (*Dermacentor variabilis*) and the lone star tick (*Amblyomma americanum*). All ticks were received by Public Health Ontario laboratories and identified morphologically using existing identification keys (e.g., Lindquist *et al.* 2016 [32]). Given the long-term goal of developing a smartphone application, camera phones (iPhone 5s, 6) were used for image acquisition. The phones were mounted 8 centimeters above the ticks which were placed on a white paper. In total, 12,588 images were captured, 2 per tick, one dorsal and one ventral. Moreover, in order to improve the quality of our data set, 1000 high-resolution tick images were taken with a camera mounted on the laboratory stereomicroscope. Our data set included 6,294 distinct ticks, of which 41% were blacklegged, and 59% were non-blacklegged ticks. A spread of fully engorged, slightly engorged, unfed, and nymph-stage ticks were included in our dataset. All tick images were manually annotated by an expert at Public Health Ontario. Fig. 1 shows a sample of tick images in the data set.

### C. Models and Training Frameworks

In this work, the automated identification of blacklegged ticks is framed as a binary classification task where a CNN model is trained to predict class labels for given tick images through the following training strategies:

1) **Training the CNN models from scratch with random initialization where all layers were open to be tuned during training.** In this setting, two CNN architectures were used including Inception-Resnet [33] and a lighter CNN model designed for this study. The lighter CNN model comprised 7 convolution layers followed by a dropout or batch normalization. In addition, average pooling layers were used to reduce the number of parameters. In total, the network had 13 layers with 5,350,633 trainable parameters out of 5,352,041 parameters (more details of the network is shown in Appendix A).
2) **Transfer learning from an Inception-Resnet CNN network pre-trained on ImageNet.** In this setting, two sets of experiments were conducted, including opening all the CNN layers to be tuned and unfreezing only the last five layers during training.
3) **Attention transfer from an Inception-Resnet [33] teacher pre-trained on ImageNet.** In this setting, the knowledge is transferred to the student network which was the lighter CNN model with and without LSR.

**FIGURE 2.**
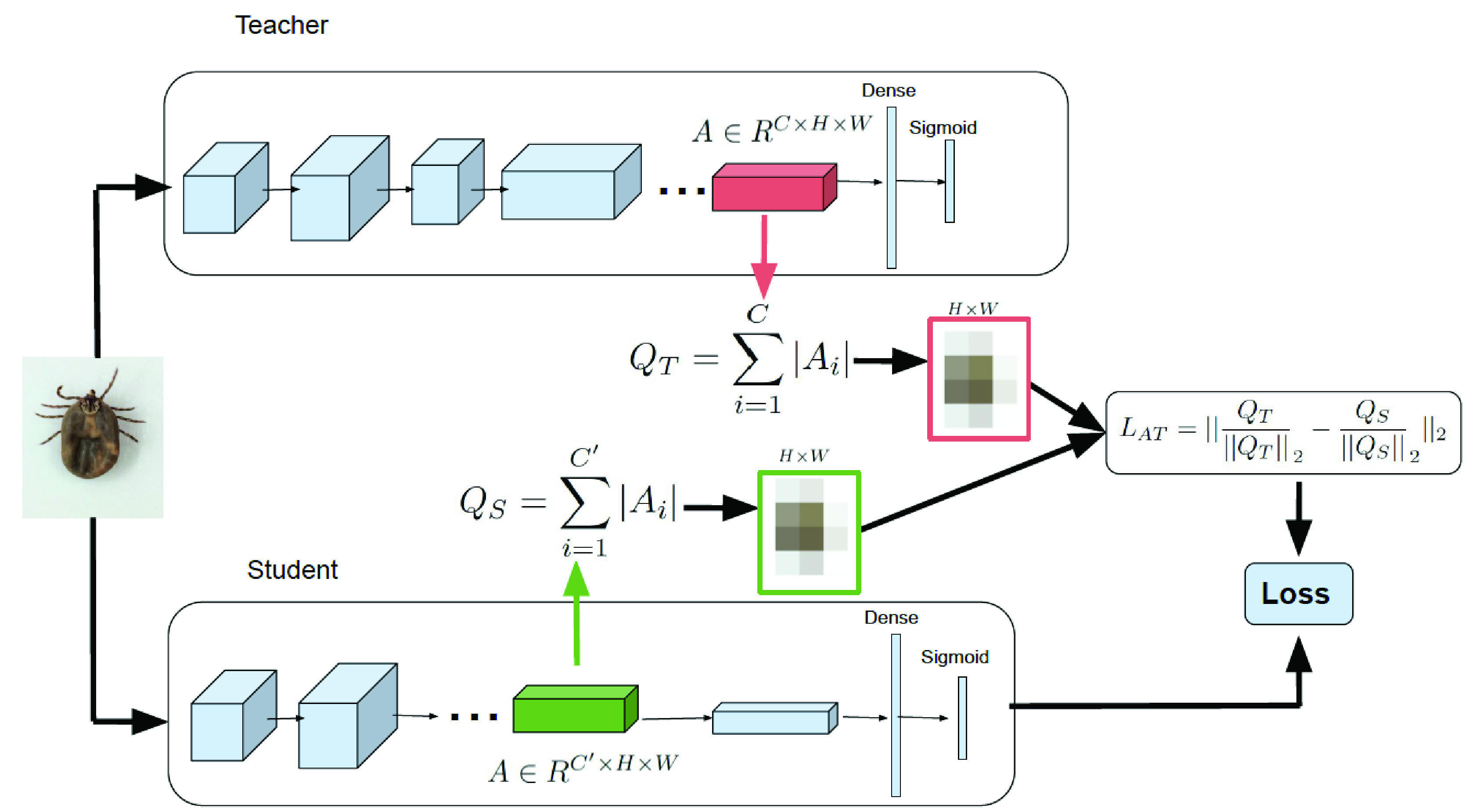
An overview of the attention transfer loss in a teacher-student learning setting. The spatial attention map is constructed by taking the sum of absolute values of a layer’s 3D tensor, A, across the channel dimension. In this setting, knowledge is transferred from the last layer of the teacher network to the one before the last layer of the student network. In the shown example, the spatial attention map $Q\in R^{H\times W}$ is $8\times 8$ and teacher’s (C) and student’s (C’) channel dimensions are 1536 and 32, respectively.

**Attention transfer:** Following the work of Zagoruyko *et al.* [29], we built an activation based attention transfer to transfer knowledge from the last layer of the teacher network (Inception-Resnet) to the one before the last layer of the student network (lighter CNN) as shown in Fig. 2. The knowledge to be transferred in our setting is a spatial attention map, constructed by taking the sum of absolute values of a layer’s 3D tensor $A\in R^{C\times H\times W}$ across the channel dimension:

\begin{equation*} Q=\sum \limits _{i=1}^{C}{|A_{i}|},\tag{1}\end{equation*}

where $C$, $H$, and $W$ are channel dimension, height, and width of a CNN layer’s tensor, A, respectively. The spatial attention map, $Q$, is therefore a 2D tensor $Q\in R^{H\times W}$. Using $l_{2}$ normalization, we calculated attention transfer loss between the teacher’s and student’s spatial attention map of the same resolution (same $H$ and $W$) as follows:

\begin{equation*} L_{AT}=||{\frac {Q_{T}}{||Q_{T}||}_{2} -\frac {Q_{S}}{||Q_{S}||}_{2}}||_{2},\tag{2}\end{equation*}

where $Q_{S}$ and $Q_{T}$ are the vectorized form of student’s and teacher’s spatial attention maps. The overall approach is shown in Fig. 2.

**Label Smoothing Regularization (LSR):** In this work, we made use of LSR as a regularization technique to smooth the loss function. For this approach, we trained two student networks where one of the students, student_1_, was trained on a subset of training data using attention transfer loss. After student_1_ was trained, it was used to generate soft labels for the entire training data as follows:

• For correctly classified images, the network produced class probabilities by converting the logits, $\theta _{i}, i\in \{0,1\}$, computed for each class, into a probability $p_{i} = \frac {1}{1+exp^{-\theta _{i}/T}}$, as suggested in [34]. $T$ is a temperature where a higher value for $T$ produces a softer probability distribution over classes.
• For incorrectly classified images, the network replaced class probabilities, $p_{i}$, with a constant probability sampled from a uniform distribution. In this work, we chose to replace the predicted probabilities for true classes with 0.6.

The second student network, student_2_, is therefore trained with the following loss function, which is a weighted combination of attention transfer and LSR:

\begin{equation*} L_{tot}= -\frac {1}{\beta }_{1} \sum \limits _{i=0}^{1}{(p_{i}\log {q_{i}})}+ \frac {1}{\beta }_{2}L_{AT}, \tag{3}\end{equation*}

where $p_{i}$ is the soft label produced by student_1_, $q_{i}$ is the output probability predicted by student_2_, and $\beta _{1}$ and $\beta _{2}$ are the weights balancing attention loss and cross-entropy loss.

### D. Web Application Development

**FIGURE 3.**
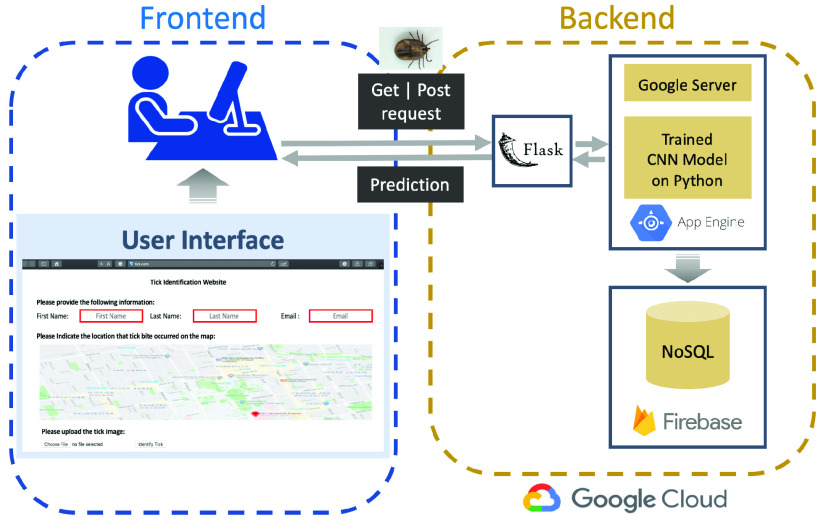
The system architecture of deploying our CNN model on the web application for early identification of blacklegged ticks. For the frontend, HTML (HyperText Markup Language) and CSS (cascading style sheets) were used to create the user interface (UI). On the backend, Python Flask application was developed to handle the get and post requests between UI and compute engine. Our trained CNN model was deployed on the app engine of the google cloud platform. The users’ data were stored in the firebase realtime database (NoSQL) as JSON and synchronized in real-time to every connected user.

As a second step toward our main objective, we created a web application that was shared internally with Public Health Ontario laboratory technicians for external validation of the model in the identification of blacklegged ticks. Using the web application, the laboratory technician can upload the image of a tick taken by a cell phone and receive feedback from the platform in less than a minute. It also captures the geolocation of the exposure and pairs it with public health data. This capability enables the assessment of the risk of Lyme disease infection and the need for prophylaxis treatment in future once the web application is open to public. Fig. 3 shows the end-to-end deployment of the CNN model as a web application. The uploaded data is processed in the backend on the compute engine of the google cloud and results will be provided to users.

## III. Results

In this section, the classification results obtained by applying different CNN models on the tick data set are presented.

For model development and evaluation, our data set was divided into a train/test split with a ratio of 11/1 without any overlap. Therefore, 12,554 images (41% blacklegged) were used for the training set, and 1034 (41% blacklegged) were used for the test set to validate the performance of the developed model. The training data was augmented with random rotation of 0°–360°, horizontal flip, vertical flip, and zoom range of 0.5–2 $\times$. Adam was used to optimize the loss function in all of the experiments. Cross validation (k=3-fold) was used for hyper-parameter tuning. The input image sizes for the lighter CNN model and Inception-Resnet network were $300 \times 300$ and $299 \times 299$, respectively. The lighter CNN model was trained for maximum 256 epochs with an initial learning rate of $10^{-3}$ and a batch size of 64. For the attention transfer approach, the classification loss was the combination of $L_{AT}$ and binary cross entropy loss. For the attention transfer+ LSR approach, the loss parameters (eq.3), including $\beta _{1}$, $\beta _{2}$, and T, were set to be 1, 2, and 5, respectively.

Table 1 reports the results of our first experiment, where the performance of training the lighter CNN and standard Inception-Resnet [33] models are compared. The lighter CNN was trained from scratch with random initialization while the standard Inception-Resnet was trained through transfer learning using ImageNet weights in addition to the random initialization. For the transfer learning, we conducted two tests where in one setting all layers were unfrozen to be trained translating to $53~{m}$ trainable parameters, and in the other setting, the last five (5) layers were fine tuned translating to only $4.5~{m}$ trainable parameters. As the results of our first experiment (Table 1) indicate, training the Inception-Resnet model either from scratch with random initialization or from ImageNet pre-trained weights without any frozen layers have the highest performances on accuracy, area under the ROC curve, and area under the precision-recall curve. We can also observe from the results that the lighter CNN obtained comparable results to both of Inception-Resnet CNN models. So, the initial layers of the network should be included and unfrozen during training the model as fine-tuning just the last layers of the CNN network on tick images perform very poorly.

**TABLE 1:** The Performance of Using Different Strategies Including the Network Size and Initialization for Training CNN Classifiers to Differentiate Between the Two Common Tick Species; Blacklegged vs Dog Ticks. The Best Performances Per Each Column are in Bold and the Second Best Scores are Underlined. ROC-AUC is the Area Under the ROC Curve and PR-AUC is the Area Under the Precision Recall Curve. Regardless of Initialization, CNN Models With Larger Number of Trainable Parameters Perform Better on Tick Data Set. The CNN Classifier Performs Very Poorly if the Initial Layers are Fixed During Training. * Only the Last 5 Layers of the Inception-Resnet Were Fine Tuned While the Rest of the CNN in the Table Were Trained From Scratch Without Any Frozen Layers

Model	Initialization	# Trainable Parameters	Accuracy	ROC-AUC	PR-AUC
lighter CNN	Random	$5.3~m$	91.68 ± 0.25	97.55 ± 0.34	95.43 ± 0.46
Inception-Resnet	Random	$53~m$	92.04 ± 0.48	98.52 ± 0.28	96.80 ± 0.99
Inception-Resnet*	ImageNet	$4.5~m$	42.10 ± 0.37	57.85 ± 0.08	47.96 ± 0.20
Inception-Resnet	ImageNet	$53~m$	91.75 ± 0.06	98.51 ± 0.38	96.77 ± 0.89

**FIGURE 4.**
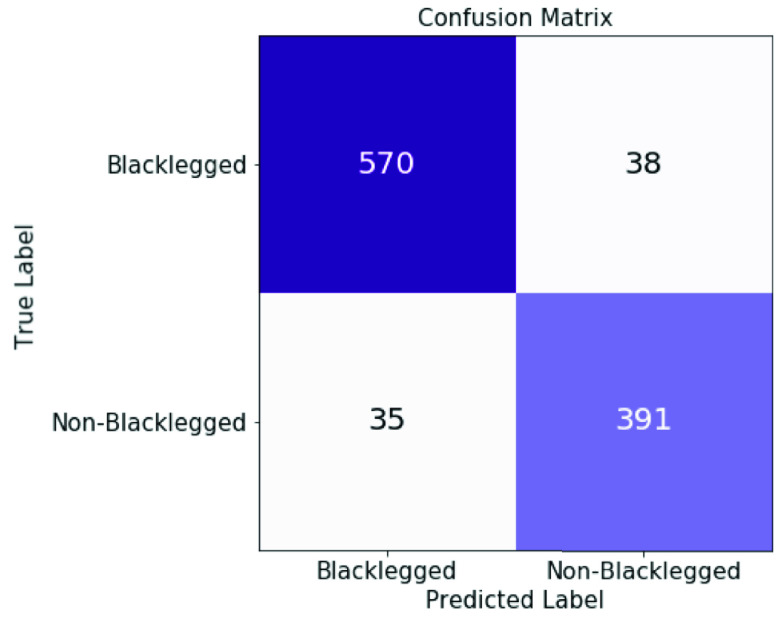
Confusion matrix for the best performing model on the tick images included in the testset. The best performing model is the combination of attention transfer and label smoothing regularization.

In our second experiment, we examined attention transfer and attention transfer+ LSR techniques from a teacher network to a student network as shown in Table 2. As explained in section II-C, two student networks were trained for attention transfer+ LSR where one student network generates soft labels. As the results indicate both attention transfer and attention transfer+ LSR models performed the same across all measures. Comparing all CNN models from Table 1 and Table 2 together, we can observe that knowledge transfer approach (Table 2) outperforms training CNN from scratch with random initialization (Table 1) based on test accuracy. However, all models achieve comparable performance on the area under the ROC curve and the area under the precision recall curve. The confusion matrix of the best model (attention transfer+ LSR) is shown in Fig. 4.

**TABLE 2:** The Performance of Using Attention Transfer and Attention Transfer With Label Smoothing Regularizer (Attention Transfer+ LSR) for Classification of Blacklegged Ticks Versus Other Tick Specious. Teachers are Inception-Resnet Pre-Trained on ImageNet, and Students are Lighter CNN Model With $5.3~m$ Trainable Parameters. The Best Performances Per Each Column are in Bold. Smoothing the Loss Function Through LSR Approach Makes the CNN Model Perform Slightly Better on Accuracy Measure

Model	Accuracy	ROC-AUC	PR-AUC
attention transfer	91.20 ± 0.33	97.70 ± 0.29	96.69 ± 0.08
attention transfer+ LSR	92.55 ± 0.39	97.32 ± 0.32	96.17 ± 0.05

**TABLE 3:** The Architecture of Lighter CNN

Layer	Number of filters, n	Size/stride	Activation function	Output size
Input	N/A	N/A	N/A	$3 \times 300 \times 300$
Convolutional	64	8/2	N/A	$64 \times 147 \times 147$
Batch normalization	N/A	N/A	Leaky Relu	$64 \times 147 \times 147$
Convolutional	128	8/1	N/A	$128 \times 140 \times 140$
Batch normalization	N/A	N/A	Relu	$128 \times 140 \times 140$
Average pool	N/A	4/2	N/A	$128 \times 69 \times 69$
Dropout	N/A	N/A	N/A	$128 \times 69 \times 69$
Convolutional	256	8/1	N/A	$256 \times 62 \times 62$
Batch normalization	N/A	N/A	Relu	$256 \times 62 \times 62$
Convolutional	128	8/1	N/A	$128 \times 55 \times 55$
Batch normalization	N/A	N/A	Relu	$128 \times 55 \times 55$
Average pool	N/A	4/2	N/A	$128 \times 26 \times 26$
Dropout	N/A	N/A	N/A	$128 \times 26 \times 26$
Convolutional	64	8/1	N/A	$64 \times 19 \times 19$
Batch normalization	N/A	N/A	Relu	$64 \times 19 \times 19$
Convolutional	32	5/2	N/A	$32 \times 8 \times 8$
Batch normalization	N/A	N/A	Relu	$32 \times 8 \times 8$
Convolutional	32	5/1	N/A	$32 \times 4 \times 4$
Batch normalization	N/A	N/A	Relu	$32 \times 4 \times 4$
Flatten	N/A	N/A	N/A	512
Fully connected	32	$512 \times 32$	Relu	32
Fully connected	4	$32 \times 4$	Relu	4
Output layer	N/A	$4 \times 1$	Sigmoid	1

**FIGURE 5.**
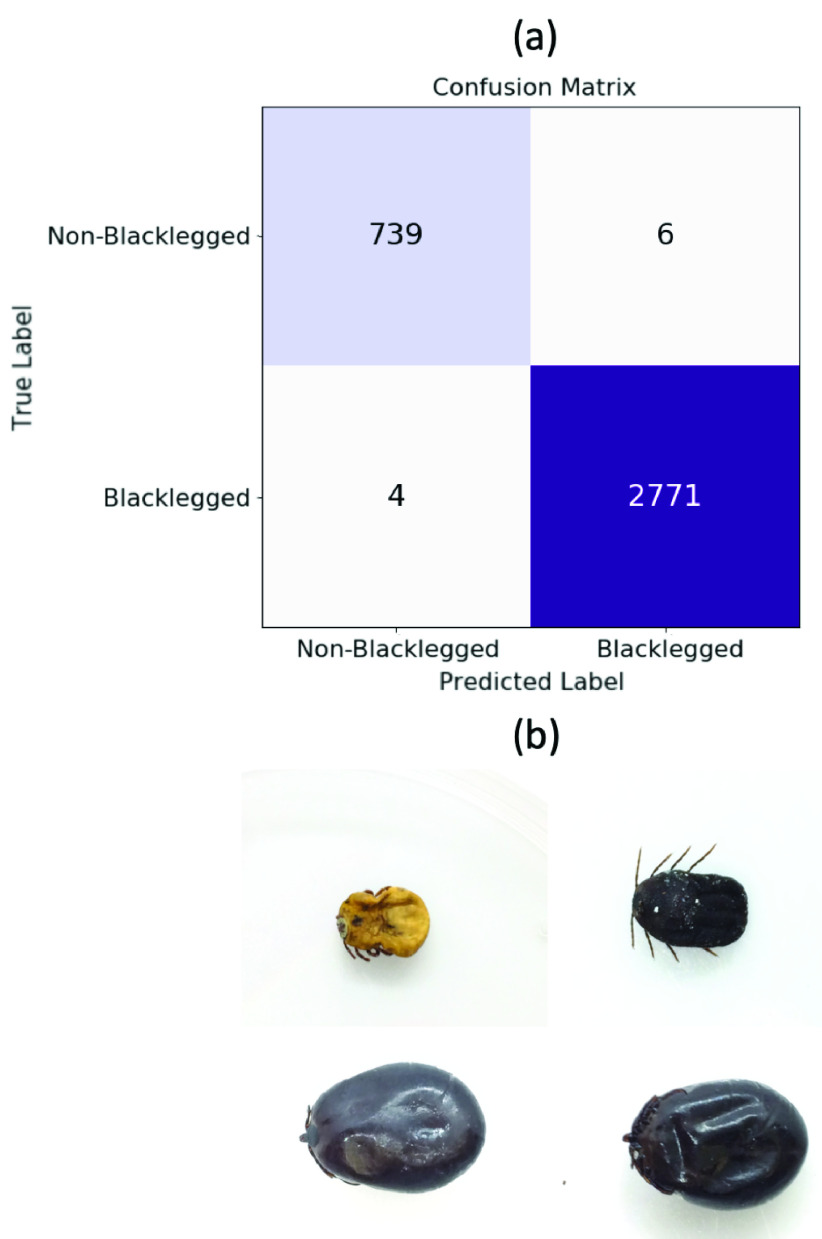
(a) Confusion matrix for the web application validation. (b) Tick images misclassified by the web application.

For validating our tool as a web application, Public Health Ontario used our service to identify 3,520 ticks (2,775 blacklegged ticks) submitted by the public from September 2020 through October 2021 in addition to routine lab identification. Expert laboratory technicians took images of the ticks and uploaded them to our web application. They then conducted a pairwise comparison between the ground truth labels from expert identification (routine lab identification) and the predicted label by using our web application. In this comparison, the web application obtained an accuracy of 99.7%, a sensitivity of 99.2%, a precision of 99.5%, and an f1-score of 99.3%. As shown in Figure 5, only 10 out of total 3,520 tick images were misclassified.

## IV. Discussion

We demonstrated that deep learning-based computer vision models are effective for the classification of blacklegged ticks versus other tick species. Our best classification model was able to identify blacklegged ticks with 92% accuracy using attention transfer and LSR techniques. In this setting, a small CNN model receives knowledge from a large CNN model and learns to behave like a large network during classification. As shown in our experiments, not only did our model outperform other models (in terms of accuracy) but also it showed a potential to be deployed on small devices such as mobile phones due to the small size of the network ( $5.3~{m}$ trainable parameters). In addition, our model will provide faster identification of blacklegged ticks (almost real time) compared to eTick.ca (within 48 hours) or identification from passive surveillance (up to 3 weeks or longer) [12]. To show the impact of our data augmentation in improving the generalization and performance of the presented models, we trained the lighter CNN model without data augmentation. This model obtained an accuracy of 53.03% ± 9.99%, a ROC-AUC of 50.08% ± 0.14%, and a PR-AUC of 41.24% ± 0.07%. This result shows a significant performance drop in comparison to the lighter CNN model with data augmentation.

To the best of our knowledge, this is the first study using deep learning and computer vision to classify blacklegged ticks from non-blacklegged ticks. Although our approach was validated on our test set and external website data, a limitation of this study was data collected in Ontario with the same camera setup and model (iPhone 6). Remedying this limitation will involve validating our approach on another dataset collected under different conditions, for example, using other phones in a different setup and various backgrounds and collecting images from different countries. Another limitation of this study is the binary classification of the ticks (blacklegged tick vs. non-blacklegged tick). With additional tick submissions going forward, this model will be further refined in the context of a greater number of non-*Ixodes*/*Dermacentor* ticks. Another limitation is that this study is the first attempt to use artificial intelligence to identify tick species, so there is no baseline (papers) for comparison. Lastly, while we are comparing this to gold standard laboratory identification of ticks, there is always a risk that a tick could be misidentified in the lab.

Deep learning and computer vision have been developed to identify a variety of agricultural pests throughout the world; for example, they have been used to identify olive fruit flies in Greece (*Bactrocera oleae*) [35]–[38]. While developed widely in the field of agricultural entomology, recently, researchers have used deep CNNs in the field of vector-borne diseases. Several studies have investigated mosquito identification [39]–[41], e.g., Park *et al.* (2020) reported a 97% classification accuracy of 8 mosquito species [42]. To overcome expertise to identify triatomine vectors of the parasite *Trypanosoma cruzi* (agent of Chagas disease), Khalighifar *et al.* (2019) were able to identify Mexican species (n = 12) and Brazilian species (n = 39) at an accuracy rate of 83.0% and 86.7%, respectively [43]. Pfieffer and Valdenegro-Toro (2020) reported a accuracy rate of 80.2% in classifying tick-borne disease skin lesions using deep learning [44]. Yang *et al.* (2015) used a support vector machine classifier with a radial basis kernel function to identify insects based on wing outlines. They reported an identification accuracy of 87% to 100% for different species. However, they did not test their approach on other insect groups (non-owlfly wings) [45]. Deep learning and computer vision have the potential to change how we study insect and tick ecology, along with vector-borne disease epidemiology. Høye *et al.* (2021) noted that besides insect identification, deep learning and computer vision could transform population monitoring of insects, including estimating insect abundance and biodiversity [46].

Future work will include public release of the web application and its further extension into a mobile app. The mobile app will allow users to receive an identification in real-time following uploading their tick picture as well as the geographical location of tick. The public release of the application will help with widespread monitoring of the distribution and relative abundance of blacklegged ticks. This method is part of a suite of techniques for blacklegged tick surveillance. Specifically, it could alleviate logistical pressures on experts and allow them to focus on other areas of tick identification and surveillance. The power of the tool will increase as the number of users uploading information increases. The ability of the tool to detect emerging blacklegged tick population will help healthcare and public health professionals in raising awareness of Lyme disease in specific regions. Currently, we are evaluating the web application in close partnership with public health experts.

We provided preliminary evidence that advanced deep learning technologies hold promise for improving blacklegged tick surveillance. In addition, there is opportunity to further refine the technology to classify other species of ticks. However, how these technologies will be adopted into an affordable, sensitive, specific, and user-friendly tool for end-users requires further examination. Our current and future work will provide insight to those interested in advancing and adopting deep learning models in the field of infectious disease surveillance and diagnostics.

## V. Conclusion

For clinicians, assessing a patient’s exposure to infectious blacklegged ticks is a critical step toward determining their risk of Lyme disease. Advanced deep learning technologies will help healthcare and public health officials monitor the geographic emergence and establishment of blacklegged ticks and their associated pathogens. Furthermore, our tool simplifies tick identification, in contrast to time-consuming and labor-intensive laboratory approaches for tick identification. This is the first report of using deep learning technologies to classify ticks, providing the basis for automation of tick surveillance and advancing tick-borne disease ecology and risk management.

## Appendix

Supplementary details about the lighter CNN model; the network has 13 layers with 5,350,633 trainable parameters composed of convolution layers and average pooling layers.
